# The *Enterococcus* secretome inhibits the growth of *Mycobacterium tuberculosis* complex mycobacteria

**DOI:** 10.1099/acmi.0.000471.v3

**Published:** 2023-06-23

**Authors:** Wafaa Achache, Jean Louis Mege, Mustapha Fellag, Michel Drancourt

**Affiliations:** ^1^​ Aix-Marseille Univ, IRD, AP-HM, MEPHI, Marseille, France; ^2^​ IHU Méditerranée Infection, Marseille, France

**Keywords:** *Enterococcus mundtii*, *Mycobacterium tuberculosis*, anti-tuberculosis activity, inhibition spectrum, tuberculosis

## Abstract

*
Enterococcus mundtii *, a commensal intestinal bacterium, was demonstrated to inhibit the growth of some *Mycobacterium tuberculosis* complex (MTC) species that cause tuberculosis in humans and mammals. To further explore this preliminary observation, we cross-investigated five *E. mundtii* strains and seven MTC strains representative of four MTC species using a standardized quantitative agar well diffusion assay. All five *E. mundtii* strains, calibrated at 10 MacFarland, inhibited the growth of all *M. tuberculosis* strains with various susceptibility profiles, but no inhibition was observed with lower inoculums. Further, eight *E. mundtii* freeze-dried cell-free culture supernatants (CFCS) inhibited the growth of *M. tuberculosis *, *Mycobacterium africanum, Mycobacterium bovis* and *Mycobacterium canettii*, the most susceptible MTC species (inhibition diameter 25±1 mm), proportionally to CFCS protein concentrations. The data reported here indicate that the *E. mundtii* secretome inhibited growth of all MTC species of medical interest, which broadens previously reported data. In the gut, the *E. mundtii* secretome may modulate the expression of tuberculosis, exhibiting an anti-tuberculosis effect, with some protective roles in human and animal health.

## Data Summary

The authors confirm that all data and protocols are provided in this article. All strains investigated in this study were obtained from The Rickettsiae Unit Strain Collection (CSUR), under the international collection number: WDCM 875.

## Introduction

Tuberculosis is a deadly infection in mammals and humans, transmissible by the transcutaneous, digestive and especially respiratory tract routes, caused by any of the 13 closely related species of mycobacteria forming the *Mycobacterium tuberculosis* complex (MTC) [1]. Among these species, *Mycobacterium tuberculosis, Mycobacterium bovis* and its derivative, *M. bovis* Bacillus bilié de Calmette-Guérin (BCG), are the most frequently encountered species in clinical microbiology laboratories worldwide [2], and *Mycobacterium africanum* and *Mycobacterium canettii* is are mainly documented in patients exposed in the Horn of Africa [3, 4]. Of particular concern are cases of tuberculosis caused by MTC isolates resistant to first-line rifampicin-based anti-tuberculosis combinations, stimulating efforts to evaluate new drugs, as well as the reintroduction of old drugs, such as those used for decades as anti-leprosy drugs [5, 6].

Over the last decade, several lines of observation have indicated reciprocal interactions between the gut microbiota and MTC mycobacteria [7–9]. MTC mycobacteria can be found in intestinal tissues and faeces in the case of intestinal tuberculosis, and in the faeces in the case of pulmonary tuberculosis [10]. Accordingly, excreted faeces could be used as surrogate specimens on which to base the detection of *M. tuberculosis* by PCR and culture [11]. Experimental models incorporating either *M. canettii* or *M. tuberculosis* have confirmed lymphatic and pulmonary tuberculosis after an oral route of transmission of these MTC pathogens [12]. Finally, these experimental data agree with medical observations made during the so-called Lübeck disaster in Germany in 1929–1933, where 251 neonates were vaccinated by the oral route with a BCG vaccine contaminated with *M. tuberculosis *, 173 neonates developed tuberculosis and 72 died [13]. Increasingly, gut microbiota distortions are being implicated in the pathogenesis of several infectious and non-infectious diseases. Therefore, the role of gut microbiota in modulating the natural history of tuberculosis is being increasingly considered, yet is not entirely understood [14]. A few recent studies have disclosed significant differences in gut microbiota composition between tuberculosis and non-tuberculosis patients: in tuberculosis patients, pathogenic species of *Actinobacteria* and *Proteobacteria* were significantly more abundant, while faeces collected from apparently healthy individuals were enriched in *Bacteroidetes* and *Firmicutes* [15, 16].

Bacteria of the genus *Enterococcus* are among *Firmicutes* commensals of the gastrointestinal tract [17] known to produce antimicrobial peptides [18–20]. In fact, *Enterococcus* species produce a wide range of diverse antimicrobial peptides, often more than one per strain, some of which are atypical and distinct from known antimicrobial peptides [21]. Enterococci have received increased attention in recent years, due to their applications in medical treatments and their consideration as beneficial organisms for health [17, 22].

Recently, we reported the *in vitro* inhibitory effect of *Enterococcus mundtii* against MTC species [23]. Here, we further investigated the experimental anti-tuberculosis activity of different *Enterococcus* species to characterize and explore the inhibition spectrum of the *Enterococcus* secretome against some MTC representative species.

## Methods

### *Mycobacterium* strains

All bacterial strains investigated were obtained from the CSUR (The Rickettsiae Unit Strain Collection) of the University-Hospital Institute laboratory (IHU-Méditerranée Infection, Marseille, France). The identification of all strains was confirmed by matrix-assisted laser desorption ionization/time of flight (MALDI-TOF) MS [24]. All manipulations of live MTC mycobacteria were performed in the NSB3 laboratory. Seven MTC strains, including four *M. tuberculosis* (strain S10, CSUR Q3640 lineage 4.8; strain S14, CSUR Q3641 lineage 4.8; strain S15, CSUR Q3643 lineage 3.1; and strain S16, CSUR Q3644 lineage 4.6)*, M. canettii* (CSUR Q3751)*, M. africanum* (CSUR 2018–2-B6) and *M. bovis* (CSUR Q0209), were inoculated in Middlebrook 7H10 agar medium (Becton Dickinson) containing 10 % oleic acid–albumin–dextrose–catalase (OADC; Becton Dickinson) for 15 days at 37 °C in an aerobic atmosphere enriched with 5 % CO_2_. Mycobacterial colonies were resuspended in 1 ml of Dulbecco’s PBS (DPBS; Thermo Fisher Scientific), vigorously mixed on a vortex mixer for 10 min, homogenized with glass beads (Sigma-Aldrich) to remove clumps and finally calibrated at 1 MacFarland using optical densitometry.

### *Enterococcus* strains

A total of 27 *Enterococcus* species were investigated in this study. These strains were grown on Columbia agar solid medium, supplemented with 5 % sheep blood (COS; bioMérieux) for 24 h in an aerobic atmosphere enriched with 5 % CO_2_ at 37 °C. *E. mundtii* colonies were suspended in 1 ml of DPBS, vortexed for 2 min and adjusted to 10 McFarland equivalent of 3×10^9^ c.f.u. ml^–1^ according to serial dilution methodology (Table 1).

**Table 1.** List of *Enterococcus* species investigated in this study from CSUR, under the international collection number: WDCM 875

CSUR international collection no. WDCM 875			
Strain	CSUR number	Strain	CSUR number
* E. mundtii *	CSURP724	* E. pallens *	CSURP1924
* E. mundtii *	CSURQ1712	* E. pallens *	CSURP1925
* E. mundtii *	CSURP7988	* E. phoeniculicola *	CSURP1828
* E. mundtii *	CSURP5399	* E. phoeniculicola *	CSURP7585
* E. mundtii *	CSURP2005	* E. pseudoavium *	CSURP1213
* E. avium *	CSURQ4946	* E. pseudoavium *	CSURP2652
* E. avium *	CSURQ5146	* E. saccharolyticus *	CSURP1919
* E. avium *	CSURQ5145	* E. saccharolyticus *	CSURP1024
* E. durans *	CSURP8822	*E. sanguinicola*	CSURP1723
* E. durans *	CSURQ2511	* E. thailandicus *	CSURP3312
* E. durans *	CSURP8400	*E. timonensis*	CSURP1024
* E. casseliflavus *	CSURQ3866	* E. cecorum *	CSURQ4894
* E. casseliflavus *	CSURQ5159	* E. malodoratus *	CSURQ5750
* E. faecalis *	CSURP6215	* E. raffinosus *	CSURQ3178
* E. faecalis *	CSURP6305	*E. burkinafasonensis*	CSURQ0835
* E. faecium *	CSURP3600	* E. devriesei *	CSURP0494
* E. faecium *	CSURQ4911	* E. diestrammenae *	CSURP0303
* E. gallinarum *	CSURP4099	* E. eurekensis *	CSURP2018
* E. gallinarum *	CSURQ4127	* E. gilvus *	CSURP5799
* E. hirae *	CSURQ3681	*E. massiliensis*	CSURP7858
* E. hirae *	CSURP2034	*E. ovatus*	CSURP1924
* E. dispar *	CSURP0234	*E. ovatus*	CSURP588
* E. dispar *	CSURP2030	*E. mediterraneensis*	CSURP2034
*E. mediterraneensis*	CSURP2034	–	–

MTC, *Mycobacterium tuberculosis* complex.

### *E. mundtii* cell-free culture supernatant

*E. mundtii* strains were grown in De Man, Rogosa, and Sharpe broth (MRS broth; Sigma-Aldrich) prepared as follows: 1 litre of MRS broth supplemented with 1 ml of Tween 80 (Sigma-Aldrich) was autoclaved at 121 °C for 15 min. A 500 µl volume of *E. mundtii* suspension inoculated into 100 ml of autoclaved MRS broth was incubated at 30 °C for 18 h under aerobic conditions. Then, *E. mundtii* cultures were centrifuged for 15 min at 3200 *g* at 4 °C and the supernatant was filtered using a 0.22 µm filter (Carl Roth) in order to obtain a cell-free culture supernatant (CFCS). The CFCS was adjusted to pH 6.5 using an NaOH solution to eliminate any pH-dependent bias, and 20 ml of CFCS was lyophilized for 24 h at −80 °C. Lyophilized CFCS was resuspended in 2 ml of DPBS, then diluted with DPBS at 1:2, 1:5, 1:8 and 1:10. CFCS dilutions were inoculated on COS medium for 24 h at 37 °C in an aerobic atmosphere enriched with 5 % CO_2_ to ensure their sterility. To determine the nature of inhibitory factors, *E. mundtii* CFCS was incubated with proteinase K (1 mg ml^−1^) at 56 °C for 2 h, prior to inhibition testing as above. Also, heat resistance was assessed by incubating the *E. mundtii* CFCS for 15 min at 100 °C, prior to inhibition testing as above. Protein concentration was quantified by the Qubit assay (Bio-Rad). Non-inoculated MRS broth was used as a negative control throughout the experimental process.

### Agar well diffusion assay

**Fig. 1.**
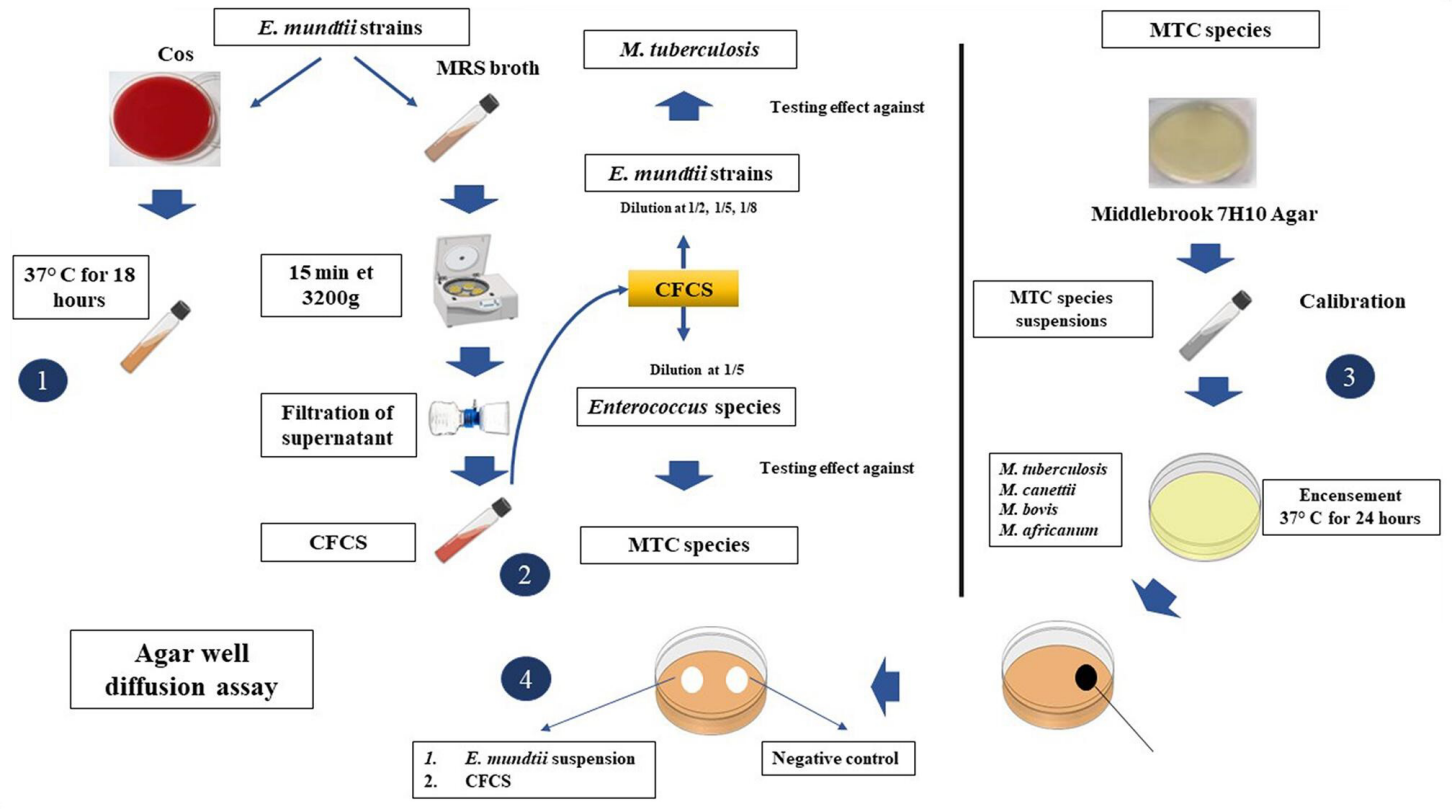
Workflow for the agar well diffusion assay used in this study. 1. *E. mundtii* suspensions. 2. *Enterococcus* species supernatants. 3. MTC species suspensions. 4. Agar well diffusion assay.

*E. mundtii* anti-tuberculosis activity was evaluated by the agar well diffusion assay. Initially, a mycobacterial suspension was inoculated on Middlebrook 7H10 agar for 24 h at 37 °C in an aerobic atmosphere enriched with 5 % CO_2_. Two wells were prepared in the centre of the 7H10 agar medium Petri dish: 100 µl of *E. mundtii* suspension was inoculated in a first well, and 100 µl of DPBS was inoculated in the other well as a negative control. Then, the anti-tuberculosis activity of *E. mundtii* CFCS was evaluated using 100 µl of CFCS in a first well, while 100 µl of MRS broth was used as a negative control in the second well. Petri dishes were incubated at 37 °C for 15 days under an aerobic condition enriched with 5 % CO_2_. The 1:2, 1:5, 1: and 1:10 CFCS dilutions were tested respectively and the diameter of MTC growth inhibition area around each well was measured in millimetres focusing restrictively on the inhibition areas. The *E. mundtii* CSURP2005 strain and its CFCS diluted at 1:5 were used to standardize the agar well diffusion assay conditions against other strains of MTC, including *M. canettii*, *M. bovis* and *M. africanum *; all manipulations were performed in triplicate (Fig. 1).

### Statistical analyses

Data were entered into Microsoft Excel for Office to calculate the mean of inhibition diameters. Statistical analysis was done using GraphPad software (v8.0). A one-way ANOVA test was used to compare the mean of the inhibition diameters of *E. mundtii* strains and to compare the susceptibility of mycobacteria to enterococci. Differences were considered statistically significant at *P*<0.05.

## Results

### *E. mundtii* suspension anti-tuberculosis activity

**Fig. 2.**
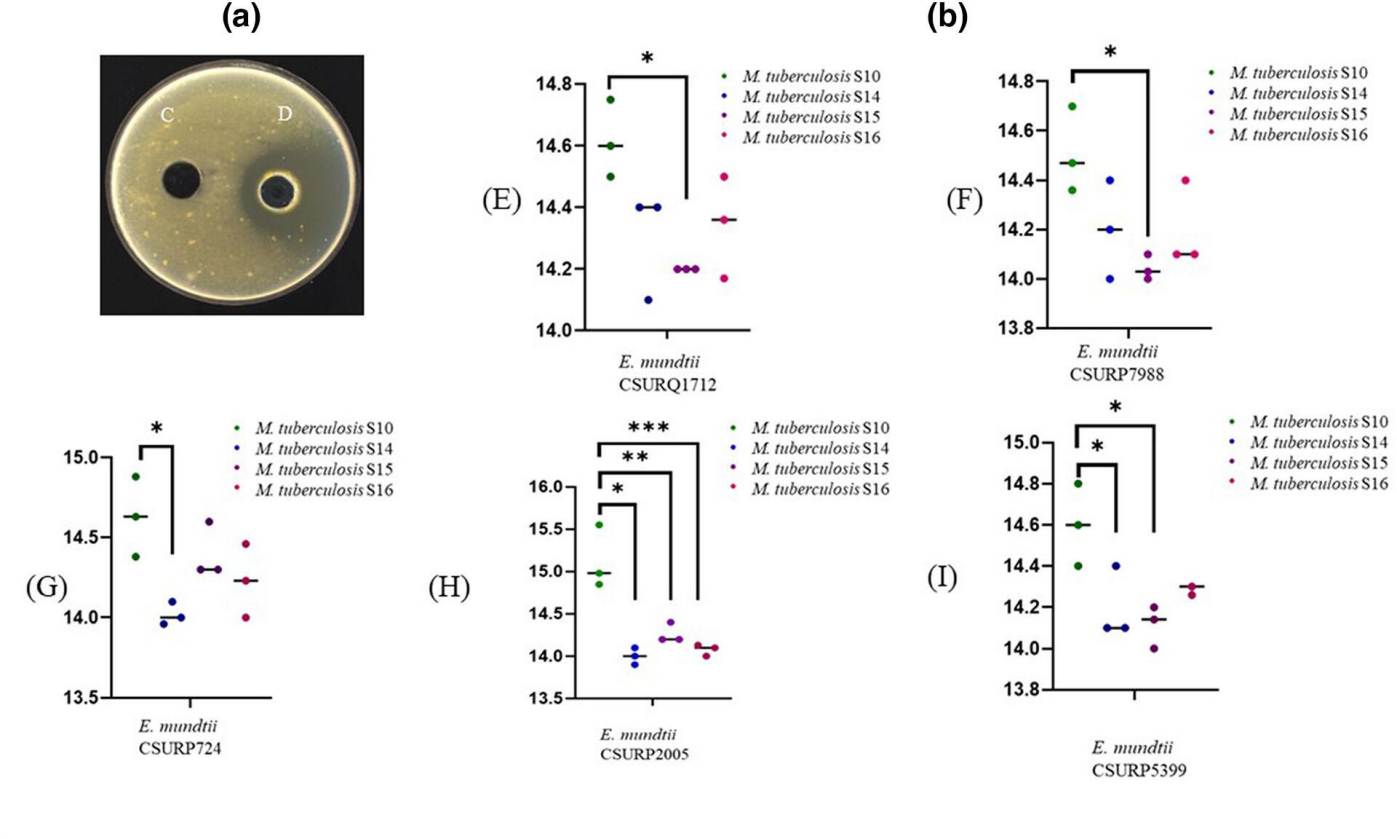
*In vitro* activity of *E. mundtii* strains against four *M. tuberculosis* strains. (a) Antimicrobial assay of *E. mundtii* strain suspensions against *M. tuberculosis* strains using the agar well diffusion assay (C, DPBS as a negative control; D, *E. mundtii* suspension). (**b)** Comparison of the means of inhibition diameters (mm) of five *E. mundtii* strains including *E. mundtii* CSURQ1712 (E), *E. mundtii* CSURP7988 (F), *E. mundtii* CSURP724 (G), *E. mundtii* CSURP2005 (H) and *E. mundtii* CSURP5399 (I) and the susceptibility of *M. tuberculosis* strain S10, *M. tuberculosis* strain S14, *M. tuberculosis* strain S15 and *M. tuberculosis* strain S16 to *E. mundtii* strains using one-way ANOVA test. Statistical significance was defined as *P<0.05.

The agar well diffusion assay indicated that the five *E. mundtii* strains evaluated here inhibited the growth of all *M. tuberculosis* strains: a clear inhibition area was observed around the well containing the 10 MacFarland inoculum of *E. mundtii* suspension after a 15 day inoculation, but not with the 8 MacFarland or 5 MacFarland suspension, suggesting that the effect observed here was dependent on the *E. mundtii* inoculum in the absence of any growth inhibition zone around the DPBS negative control (Fig. 2a). Comparing the inhibition zone diameters resulting from cross-investigation of the five *E. mundtii* strains and the four *M. tuberculosis* strains (S10, S14, S15, S16) revealed that *E. mundtii* strains had a significantly different inhibitory effect as follows: *E. mundtii* CSURQ1712 and *E. mundtii* CSURP7988 between two strains *M. tuberculosis* S10 and *M. tuberculosis* S15 (*P*<0.05) (Fig. 2bE and bF), *E. mundtii* CSURP724 between *M. tuberculosis* S10 and *M. tuberculosis* S14 (*P*<0. 05) (Fig. 2bG), *E. mundtii* CSURP5399 between *M. tuberculosis* S10 and all other *M. tuberculosis* strains tested (*P*<0.05) (Fig. 2bH), and finally *E. mundtii* CSURP2005 between three strains *M. tuberculosis* S10, *M. tuberculosis* S14 and *M. tuberculosis* S15 (Fig. 2bI). Also, *M. tuberculosis* S10, which was more susceptible, exhibited the largest inhibition area (inhibition diameter 15.5±1 mm) (Table 2).

**Table 2.** Growth inhibition of four strains of *M. tuberculosis* by five strains of *E. mundtii* and DPBS buffer as a negative control The measurement mean of growth inhibition is expressed in millimetres. Measurement of the inhibition area is in mm^2^.

Strain	DPBS	* M. tuberculosis * strain S10	*M. tuberclosis* strain S14	* M. tuberculosis * strain S15	* M. tuberculosis * strain S16
* E. mundtii * CSURQ1712	0	14.6±1 mm (167 mm²)	14.3±1 mm (160 mm²)	14.2±1 mm (158 mm²)	14.36±1 mm (161.9 mm²)
* E. mundtii * CSURP724	0	14.63±1 mm (168.1 mm²)	13.98±1 mm (153 mm²)	14.36±1 mm (161.9 mm²)	14.23±1 mm (159.3 mm²)
* E. mundtii * CSURP7988	0	14.51±1 mm (165.3 mm²)	14.2±1 mm (158 mm²)	14.13±1 mm (156.8 mm²)	14.2±1 mm (158 mm²)
* E. mundtii * CSURP5399	0	14.6±1 mm (167 mm²)	14.2±1 mm (158 mm²)	14.14±1 mm (157 mm²)	14.36±1 mm (161.9 mm²)
* E. mundtii * CSURP2005	0	15.5±1 mm (188.6 mm²)	14±1 mm (153.9 mm²)	14.3±1 mm (160.6 mm²)	14.23±1 mm (153.9 mm²)

### CFCS anti-tuberculosis activity

**Fig. 3.**
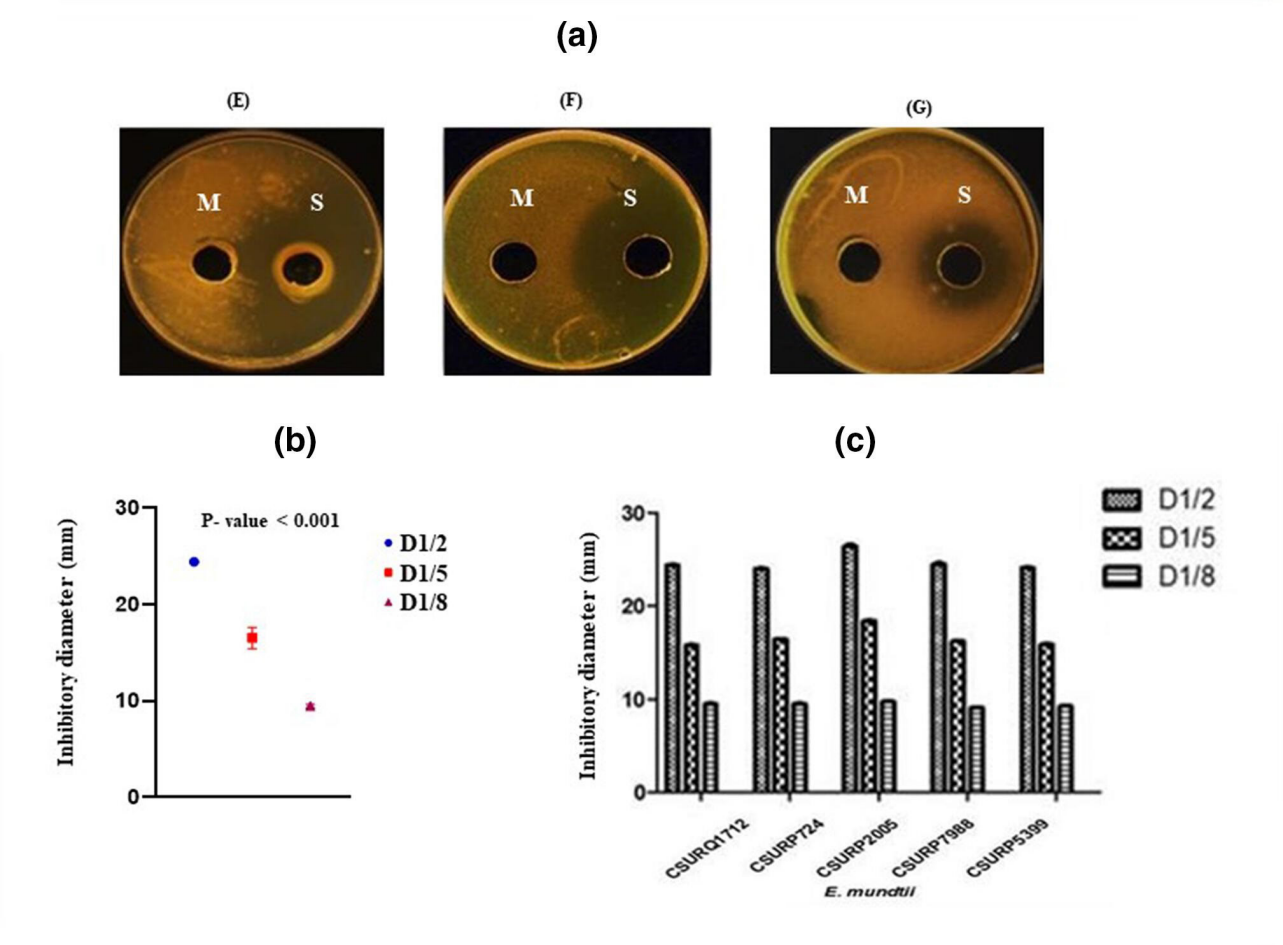
*In vitro* activity of *E. mundtii* CSURP2005 CFCS against *M. tuberculosis* S10. (a) Antimicrobial assay of *E. mundtii* CSURP2005 CFCS dilution 1:2 (E), 1:5 (F) and 1:8 (G) against *M. tuberculosis* using agar well diffusion assay (M: MRS broth; S: *E. mundtii* CSURP2005 CFCS). (b) Comparison of the means of inhibition diameters (mm) of *E *. *mundtii* strain CFCS dilutions using the Kruskal–Wallis test (one-way ANOVA). (c) Inhibition diameters of MTC strains dependent on the dilution (D) of CFCS.

**Fig. 4.**
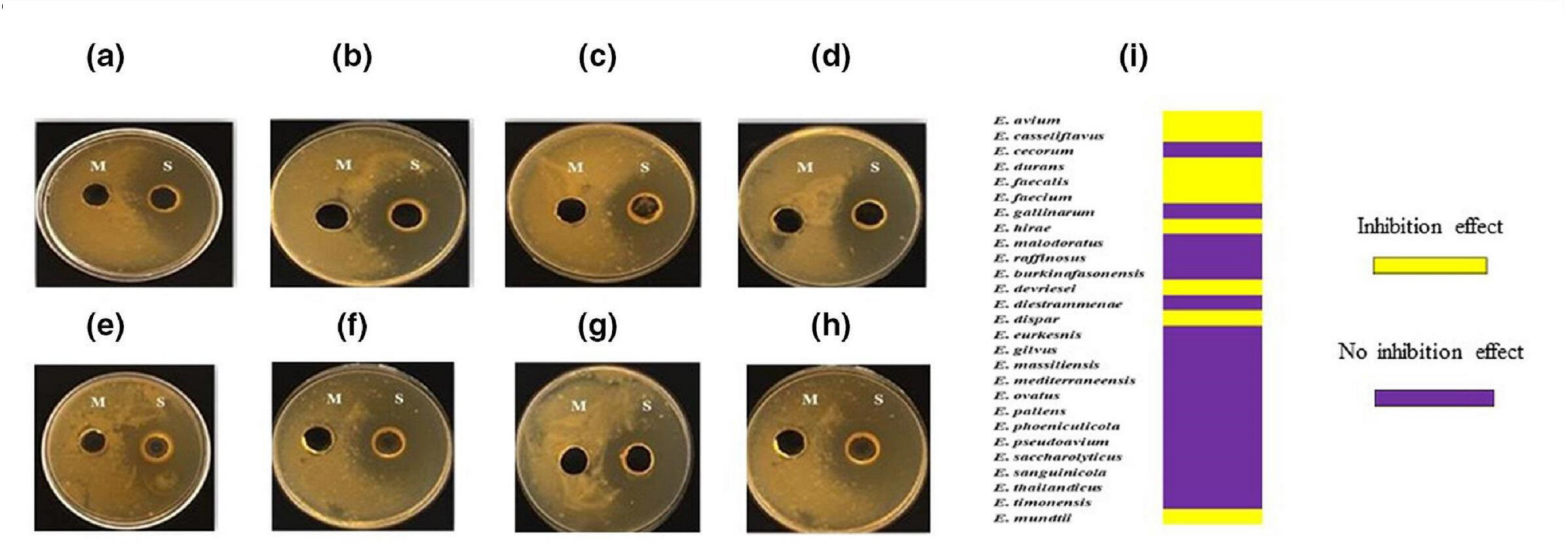
*In vitro* activity of *Enterococcus* species CFCS against *M. tuberculosis *. (a) *E. avium* CSURQ4946. (b) *E. devriesei* CSURP0494. (c) *E. durans* CSURP8822. (d) *E. dispar* CSURP2034. (e) *E. hirae* CSURQ3681. (f) *E. casseliflavus* CSURQ5159. (g) *E. faecalis* CSURP6215. (h) *E. faecium* CSURP3600 (M: MRS broth; S: *Enterococcus* CFCS). (i) Heat map summarizing inhibitory (yellow) or non-inhibitory (violet) effect of the *Enterococcus* species against *M. tuberculosis *.

**Fig. 5.**
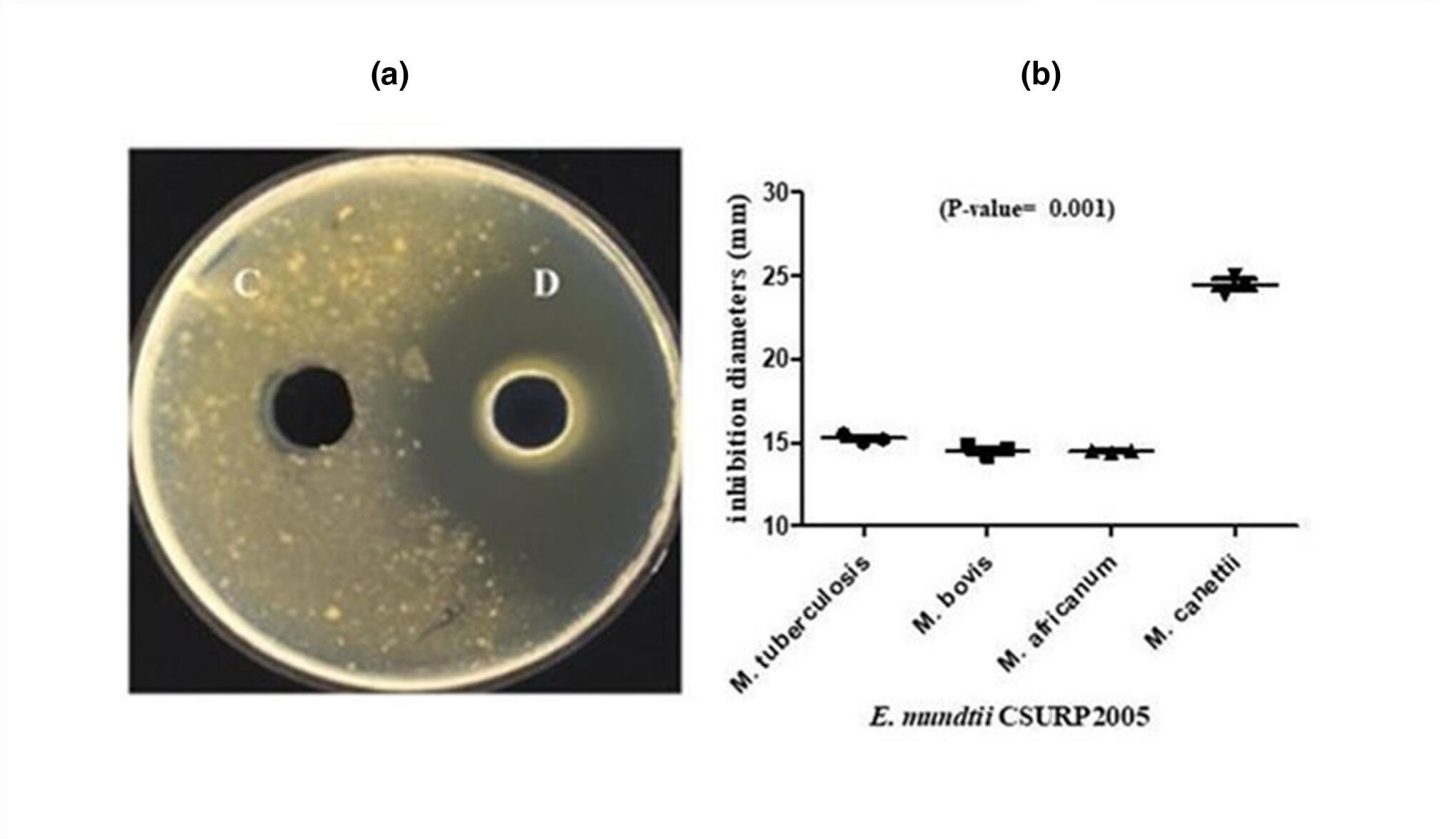
*In vitro* activity of *E. mundtii* CURPS2005 CFCS against *M. tuberculosis *, *M. africanum *, *M. bovis* and *M. canettii*. (a) Antimicrobial assay of *E. mundtii* CSURP2005 CFCS dilution 1:5 against *M. canettii* using the agar well diffusion assay. C: MRS broth; D: *E. mundtii* CSURP2005 CFCS diluted to 1:5. (b) Comparison of the means of *E. mundtii* CSURP2005 inhibition diameters (mm) against *M. tuberculosis *, *M. africanum *, *M. bovis* and *M. canettii* using the Kruskal–Wallis test (one-way ANOVA on ranks).

The protein concentration in each CFCS dilution of five *E. mundtii* strains (Table 3) indicated a maximum value for *E. mundtii* CSURP2005 (24.03 µg ml^−1^) and a minimum value for *E. mundtii* CSURP7988 (21.2 µg ml^−1^), while *E. mundtii* CSURQ1712, *E. mundtii* CSURP0724 and *E. mundtii* CSURP5399 showed values of 21.4, 22.5 and 22.3 µg ml^−1^, respectively. When tested with *M. tuberculosis* strain S10, the five *E. mundtii* strains, CFCS diluted to 1:2, 1:5 and 1:8, showed anti-tuberculosis activity, while no zone of inhibition was observed with the 1:10 diluted CFCS and the negative control in MRS broth (Fig. 3a). An area of inhibition was observed around the well containing the CFCS, which varied with CFCS concentration; the 1:8 dilution showed the lowest inhibition activity (inhibition diameter 9±1 mm). Dilution less than 1:8 showed no effect against *M. tuberculosis* strains (Table 4). A significant difference was observed between the three dilutions of five CFCS (*P*=0.001) (Fig. 3b). The inhibition diameters observed here were proportional to the CFCS fraction and the protein content of each dilution (Fig. 3c). The *E. mundtii* CSURP2005 strain exhibiting the highest protein content and its 1:5 dilution with a more representative inhibition zone was chosen to standardize the agar well diffusion assay used in this study. Treating *E. mundtii* CFCS with proteinase K resulted in total disappearance of the inhibition zones, suggesting the protein nature of the inhibitory factors. Furthermore, anti-tuberculosis activity was not affected by 15 min of heating at 100 °C. Also, from a total of 27 *Enterococcus* species, eight showed anti-tuberculosis activity against four MTC species. More precisely, 15 strains showed anti-tuberculosis inhibitory effects, including five *E. mundtii* strains (CSURQ1712, CSURP724, CSURP7988, CSURP5399 and CSURP2005), two *E. avium* strains (CSURQ4946 and CSURQ5145), two *E. durans* (CSURP8822 and CSURQ2511), one *E. casseliflavus* strain (CSURQ3866), one *E. faecalis* strain (CSURP6215), one *E. faecium* strain (CSURP3600), one *E. hirae* strain (CSURQ3681), one *E. dispar* strain (CSURP0234) and one *E. devriesei* strain (CSURP0494), with variable susceptibility between strains (Fig. 4). There was no significant difference in the susceptibility of *M. tuberculosis *, *M. bovis* and *M. africanum *, but *M. canettii* exhibited the highest susceptibility, with an inhibition area diameter of 25±1 mm (*P*=0.001) (Fig. 5).

**Table 3.** Dilution of the protein concentration (µg ml^–1^) of the CFCS of the five *E. mundtii* strains investigated here

Strains	* E. mundtii * CSURP2005	* E. mundtii * CSURP5399	* E. mundtii * CSURP724	* E. mundtii * CSURP7988	* E. mundtii * CSURQ1712
Pure	24.03	22.3	22.5	21.2	21.4
Dilution 1:2	12.01	11	11.3	11	10.7
Dilution 1:5	5	4.4	5	4.24	4.28
Dilution 1:8	3	3	2.8	2.6	2.7
Dilution 1:10	2.4	2.23	2.2	2	2

**Table 4.** Mean inhibition diameters (mm) of *E. mundtii* strains in CFCS dilutions, and MRS broth as a negative control, and measurement of the inhibition area (mm²)

Strains	MRS broth	* M. tuberculosis* strain S10 (dilution 1:2)	* M. tuberculosis* strain S10 (dilution 1:5)	* M. tuberculosis* strain S10 (dilution 1:8)	* M. tuberculosis* strain S10 (dilution 1:10)
* E. mundtii * CSURQ1712	0	24.39±1 mm (476 mm²)	15.82±1 mm (197 mm²)	9.51±1 mm (71 mm²)	0
* E. mundtii * CSURP724	0	24.02±1 mm (452 mm²)	16.43±1 mm (212 mm²)	9.52±1 mm (71.1 mm²)	0
* E. mundtii * CSURP2005	0	26.4±1 mm (547 mm²)	18.42±1 mm (266 mm²)	9.7±1 mm (73.8 mm²)	0
* E. mundtii * CSURP7988	0	24.48±1 mm (470 mm²)	16.21±1 mm (206 mm²)	9.3±1 mm (68 mm²)	0
* E. mundtii * CSURP5399	0	24.12±1 mm (457 mm²)	15.8±1 mm (196 mm²)	9.3±1 mm (68 mm²)	0

## Discussion

The fact that *E. mundtii* inhibited the growth of some MTC species had been previously described in our laboratory [23], and the data reported here broadened such previous observations by cross-incorporating a set of *E. mundtii* strains and MTC species using a standardized agar well diffusion assay to compare cross-inhibition values. Accordingly, we observed that both *E. mundtii* suspensions and derived lyophilized culture supernatants inhibited the growth of *M. tuberculosis, M. africanum, M. bovis* and *M. canettii*. This inhibition effect was reproducible and authenticated by the absence of zones of clearance. The inhibitory effect was proportional to the *E. mundtii* inoculum and the concentration of proteins in the derived lyophilized culture supernatant, while no zone of inhibition was observed with the 1:10 diluted CFCS considered as MIC, suggesting that inhibition of MTC growth was due to molecules excreted by *E. mundtii* in the culture broth, probably heat-stable peptides and proteins, most likely bacteriocins, which are indeed heat-stable antimicrobial peptides produced extracellularly and synthesized by the ribosomal pathway, encoded by the plasmid or the chromosome [25, 26].

Indeed, *E. mundtii* was acknowledged to produce bacteriocins [26–29] with antibacterial activity against various isolates of Gram-positive genera. *E. mundtii* strain ATO6 and *E. mundtii* strain NFRI 7393 isolated from vegetables produced the bacteriocin mundticin, inhibiting growth of isolates of the genus *Listeria* [30] whereas *E. mundtii* strain QU 25 isolated from ovine faeces and *E. mundtii* strain CRL35 isolated from cheeses inhibited growth of various strains of *Pediococcus, Listeria, Lactobacillus* and *Leuconostoc* [27].

In the genus *Enterococcus *, anti-mycobacterial activities of bacteriocins have been reported. *Enterococcus italicus* strain BLN34 isolated from cow’s milk inhibited growth of *Mycobacterium kansasii* [31], and *Enterococcus hirae* showed antagonistic effects against *Mycobacterium smegmatis* Mc155 [18]. Finally, *Enterococcus faecalis* has been characterized by its ability to inhibit the multiplication of *M. tuberculosis* [32].

As *E. mundtii* was detected in the gut microbiota in some individuals where *M. tuberculosis* was also detected, our data suggested that the *E. mundtii* secretome, probably bacteriocins, may modulate tuberculosis in the gut microbiota. The association between the gut microbiota and *M. tuberculosis* infection remains an avenue to be explored [33, 34]. Multiple metagenomic studies have indicated that the gut bacterial microbiota investigated in pulmonary tuberculosis patients significantly differed from that of tuberculosis-free, apparently healthy individuals [15, 35]. A decrease in the proportion of *Bacteroidetes* and *Firmicutes* has already been shown in the gut microbiota of tuberculosis patients compared to apparently healthy controls [34, 36]. Likewise, *Proteobacteria* and *Bacteroidetes* have been found to characterize the pulmonary microbiota in tuberculosis patients, while that collected in apparently healthy individuals was marked by the presence of *Firmicutes* bacteria [37].

Together, the findings of the present study support the need for additional research to investigate the potential contribution of enterococci to the control of mycobacteria.
