# Corrigendum: The Enterococcus secretome inhibits the growth of Mycobacterium tuberculosis complex mycobacteria

**DOI:** 10.1099/acmi.0.000843

**Published:** 2024-05-22

**Authors:** Wafaa Achache, Jean Louis Mege, Mustapha Fellag, Michel Drancourt

**Affiliations:** 1 Aix- Marseille Univ, IRD, AP- HM, MEPHI, Marseille, France; 2 IHU Méditerranée Infection, Marseille, France

An external reader contacted the journal to highlight that, in the published version of this article [1], Fig. 4f was a duplication of panel 4h.

The authors explained that this was a result of a mislabelling of their data, and have provided a corrected version of Fig. 4. The corrected figure is provided below:

**Figure:**
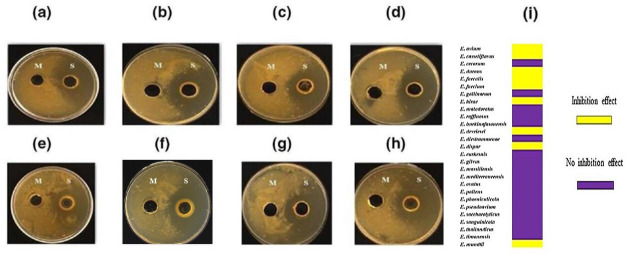


*In vitro* activity of *Enterococcus* species CFCS against *M*. *tuberculosis*. (a) *E. avium* CSURQ4946. (b) *E. devriesei* CSURP0494. (c) *E. durans* CSURP8822. (d) *E. dispar* CSURP2034. (e) *E. hirae* CSURQ3681. (f) *E. casseliflavus* CSURQ5159. (g) *E. faecalis* CSURP6215. (h) *E. faecium* CSURP3600 (M: MRS broth; S: *Enterococcus* CFCS). (i) Heat map summarizing inhibitory (yellow) or non-inhibitory (violet) effect of the *Enterococcus* species against *M. tuberculosis*.

The authors apologise for any inconvenience caused. 
